# Longitudinal Relationships Between Depressive Symptom Severity and Phone-Measured Mobility: Dynamic Structural Equation Modeling Study

**DOI:** 10.2196/34898

**Published:** 2022-03-11

**Authors:** Yuezhou Zhang, Amos A Folarin, Shaoxiong Sun, Nicholas Cummins, Srinivasan Vairavan, Rebecca Bendayan, Yatharth Ranjan, Zulqarnain Rashid, Pauline Conde, Callum Stewart, Petroula Laiou, Heet Sankesara, Faith Matcham, Katie M White, Carolin Oetzmann, Alina Ivan, Femke Lamers, Sara Siddi, Elisabet Vilella, Sara Simblett, Aki Rintala, Stuart Bruce, David C Mohr, Inez Myin-Germeys, Til Wykes, Josep Maria Haro, Brenda WJH Penninx, Vaibhav A Narayan, Peter Annas, Matthew Hotopf, Richard JB Dobson, RADAR-CNS consortium

**Affiliations:** 1 Department of Biostatistics & Health Informatics Institute of Psychiatry, Psychology and Neuroscience King's College London London United Kingdom; 2 Institute of Health Informatics University College London London United Kingdom; 3 NIHR Biomedical Research Centre at South London and Maudsley NHS Foundation Trust and King's College London London United Kingdom; 4 Health Data Research UK London University College London London United Kingdom; 5 NIHR Biomedical Research Centre at University College London Hospitals NHS Foundation Trust London United Kingdom; 6 Janssen Research and Development LLC Titusville, NJ United States; 7 Department of Psychological Medicine Institute of Psychiatry, Psychology and Neuroscience King's College London London United Kingdom; 8 Department of Psychiatry Amsterdam Public Health Research Institute and Amsterdam Neuroscience, Amsterdam University Medical Centre Vrije Universiteit and GGZ inGeest Amsterdam Netherlands; 9 Teaching Research and Innovation Unit Parc Sanitari Sant Joan de Déu Fundació Sant Joan de Déu Barcelona Spain; 10 Centro de Investigación Biomédica en Red de Salud Mental Madrid Spain; 11 Faculty of Medicine and Health Sciences Universitat de Barcelona Barcelona Spain; 12 Hospital Universitari Institut Pere Mata Institute of Health Research Pere Virgili Universitat Rovira i Virgili Reus Spain; 13 Department of Psychology Institute of Psychiatry, Psychology and Neuroscience King's College London London United Kingdom; 14 Center for Contextual Psychiatry Department of Neurosciences Katholieke Universiteit Leuven Leuven Belgium; 15 Faculty of Social Services and Health Care LAB University of Applied Sciences Lahti Finland; 16 RADAR-CNS Patient Advisory Board King's College London London United Kingdom; 17 Center for Behavioral Intervention Technologies Department of Preventive Medicine Northwestern University Evanston, IL United States; 18 South London and Maudsley NHS Foundation Trust London United Kingdom; 19 H. Lundbeck A/S Copenhagen Denmark; 20 See Acknowledgments

**Keywords:** depression, mobile health, location data, mobility, dynamic structural equation modeling, mHealth, mental health, medical informatics, modeling

## Abstract

**Background:**

The mobility of an individual measured by phone-collected location data has been found to be associated with depression; however, the longitudinal relationships (the temporal direction of relationships) between depressive symptom severity and phone-measured mobility have yet to be fully explored.

**Objective:**

We aimed to explore the relationships and the direction of the relationships between depressive symptom severity and phone-measured mobility over time.

**Methods:**

Data used in this paper came from a major EU program, called the Remote Assessment of Disease and Relapse–Major Depressive Disorder, which was conducted in 3 European countries. Depressive symptom severity was measured with the 8-item Patient Health Questionnaire (PHQ-8) through mobile phones every 2 weeks. Participants’ location data were recorded by GPS and network sensors in mobile phones every 10 minutes, and 11 mobility features were extracted from location data for the 2 weeks prior to the PHQ-8 assessment. Dynamic structural equation modeling was used to explore the longitudinal relationships between depressive symptom severity and phone-measured mobility.

**Results:**

This study included 2341 PHQ-8 records and corresponding phone-collected location data from 290 participants (age: median 50.0 IQR 34.0, 59.0) years; of whom 215 (74.1%) were female, and 149 (51.4%) were employed. Significant negative correlations were found between depressive symptom severity and phone-measured mobility, and these correlations were more significant at the within-individual level than the between-individual level. For the direction of relationships over time, Homestay (time at home) (φ=0.09, *P*=.01), Location Entropy (time distribution on different locations) (φ=−0.04, *P*=.02), and Residential Location Count (reflecting traveling) (φ=0.05, *P*=.02) were significantly correlated with the subsequent changes in the PHQ-8 score, while changes in the PHQ-8 score significantly affected (φ=−0.07, *P*<.001) the subsequent periodicity of mobility.

**Conclusions:**

Several phone-derived mobility features have the potential to predict future depression, which may provide support for future clinical applications, relapse prevention, and remote mental health monitoring practices in real-world settings.

## Introduction

Depression is a prevalent and serious mental health disorder that is a leading cause of disability worldwide [1]. It can cause physical health and psychological function problems, resulting in loss of productivity and a high social burden [2-5]. Currently, diagnosis of depression relies on skilled clinicians and self-report questionnaires, which have limitations that include subjective bias and dynamic information loss [6]. Consequently, many people with depression do not receive timely and effective treatment [7], and more efficient methods for detecting and monitoring depression are needed. Recently, the use of mobile phones with embedded sensors for depression detection and monitoring, to provide new ways for supporting both depressed people and clinicians, has been investigated [8].

We focused on exploring how phone-collected location data could link individuals’ mobility and depression. Past survey-based studies found that mobility is significantly and negatively associated with depression [9-11]. Several longitudinal survey–based studies reported a bidirectional relationship between depression and mobility over time, that is, decreased mobility worsened subsequent depressive symptoms and vice versa [10,11]. If the changes in mobility that occur before changes in depression can be captured by mobile phone technologies, early intervention can take place, which could prevent depression relapse or deterioration. Therefore, it is valuable to investigate relationships between depressive symptom severity and phone location data over time.

In recent years, there have been several studies [12-22] exploring the associations between depressive symptom severity and mobility features extracted from phone-collected location data that have shown that mobility measured by phones is negatively associated with the severity of depressive symptoms which is consistent with past survey-based studies; however, not many have explored the direction of the relationships between depression and mobility over time. Meyerhoff et al [22] recently found that phone-derived mobility features were correlated with subsequent changes in depression, but not vice versa. However, the autoregressive nature of depressive states and mobility levels [23-25] and the influence of individual differences may affect the results. In addition, the limitations of many previous phone-based studies [12-14,18-21] included relatively small and homogeneous (eg, university students) populations and the lack of comparison of between-individual and within-individual differences. To address these limitations, we aimed to explore the relationships and the direction of relationships over time between phone-derived mobility features and depressive symptom severity on a large multicenter data set.

## Methods

### Study Design

We used a large longitudinal data set of an EU research program called Remote Assessment of Disease and Relapse–Major Depressive Disorder, which explored the utility of remote measurement technologies in long-term (up to 2 years) depression monitoring [26]. We first used existing mobility features and then designed several new mobility features, which were extracted from this data set. Then, we assessed the relationships and direction of the relationships between depressive symptom severity and mobility features over time using dynamic structural equation models [27]. Furthermore, we investigated the effects of individual differences (such as demographics) on the models at the between-individual level.

### Study Participants and Settings

All participants in the study had at least one diagnosis of depression in the most recent 2 years and were recruited from 3 countries (Netherlands, Spain, and the United Kingdom); additional details descriptions are reported in [28]. Participants’ passive data (eg, location, steps, and sleep) and active data (eg, questionnaires) were respectively collected via passive remote measurement technologies and active remote measurement technologies apps provided by an open-source platform (RADAR-base) [29]. A patient advisory board comprising service users co-developed the study and were involved in the choice of measures, the timing, and issues of engagement and in developing the analysis plan.

### Ethics

Ethical approval was obtained from the Camberwell St. Giles Research Ethics Committee (17/LO/1154) in London, from the *Fundacio Sant Joan de Deu* Clinical Research Ethics Committee (CI: PIC-128-17) in London, and from the *Medische Ethische Toetsingscommissie VUms* (2018.012–NL63557.029.17) in the Netherlands.

### Phone Location and Depression Questionnaire Data

We focused on phone location data and data from the 8-item Patient Health Questionnaire (PHQ-8) [30]. The passive remote measurement technologies app measured participants’ location coordinates (longitude and latitude) using 2 providers (GPS and network sensors) periodically every 10 minutes. To protect participants’ private information, raw locations were obfuscated by adding a unique and random reference location which was assigned to each participant at the start of the study [31]. The participant’s self-reported depressive symptom severity was measured via the PHQ-8, with a score between 0 and 24 [30], which was assessed through the active remote measurement technologies app every 2 weeks (thus, the 2 weeks preceding each PHQ-8 record was the PHQ-8 interval).

### Data Inclusion Criteria

Several factors may affect our analysis, such as the COVID-19 pandemic, location data accuracy, and missing data. Notably, the COVID-19 pandemic and related lockdown policies greatly impacted European people’s mobility behaviors [32]. Therefore, according to suggestions in previous studies [6,14,16,19,33] and our experiences, we selected a subset of the data set [26] using the 3 criteria: (1) data from before February 2020 (prior to COVID-19 interventions in Europe) [6,33] were included, (2) location records with an error larger than 165 meters were removed [14,16], and (3) the amount of missing location data in a given PHQ-8 interval was limited to 50% [14,16,19].

### Data Preprocessing

We calculated the distances between consecutive location records and the instantaneous speeds at all location records. The distance between 2 consecutive location records was computed by using the Haversine formula [34]. The instantaneous speed was approximated by dividing the distance by the time between 2 consecutive location records. We regarded one location record as a stationary point if its instantaneous speed was less than 1 km/h; otherwise, we considered it a moving point [14,19].

The second procedure was location clustering. Since the density-based spatial clustering of applications with noise method [35] can treat low-density location points as outliers, avoiding overestimating the number of locations clusters [14], we used this method for location clustering, using hyperparameters and the method for handling unequal sampling intervals from [14].

### Feature Extraction

We extracted 11 mobility features (Table 1) from location data in each PHQ-8 interval (14 days), of which 4 features (3 frequency-domain features to reflect periodic characteristics of mobility and 1 feature to represent the number of temporary residential locations during the past 14 days) are new.

**Table 1:** A list of mobility features used in this study and their short descriptions.

Feature	Description
Location Variance	Variance of longitude and latitude coordinates
Moving Time	Percentage of time spent in moving
Moving Distance	Distance between all location points weighted by available time
Number of Clusters	The number of location clusters found using density-based spatial clustering of applications with noise
Location Entropy	Entropy of time distribution over different locations
Normalized Entropy	Location Entropy normalized by the number of clusters
Homestay	Percentage of time spent at home
Residential Location Count	The number of temporary residential locations
Long-term Rhythm	Percentage of frequency bins within the long-term period (>1 day) of spectrum for longitude and latitude coordinates
Circadian Rhythm	Percentage of frequency bins within the circadian period (24 hours) of spectrum for longitude and latitude coordinates
Short-term Rhythm	Percentage of frequency bins within the short-term period (<1 day) of spectrum for longitude and latitude coordinates

### Time-Domain Features

#### Location Variance

The Location Variance represented the variability of each participant’s locations [19] and was calculated as log(*Var*(*Lon*)+*Var*(*Lat*)), where log is the logarithm, and *Var*(*Lon*) and *Var*(*Lat*) represent the variances of the longitude and latitude coordinates, respectively, in one PHQ-8 interval.

#### Moving Time

The Moving Time represented the percentage of time that a participant spent in moving in one PHQ-8 interval [19]. The feature was computed by dividing the sum duration for all moving points by the sum of available time in one PHQ-8 interval.

#### Moving Distance

The Moving Distance was adjusted by dividing the total distance by the available time (in hours) in one PHQ-8 interval. In previous studies [18,19], the total distance obtained by accumulating distances between all location records; however, this total distance was affected by the missing data rate.

#### Number of Clusters

The number of the unique location clusters that a participant visited in one PHQ-8 interval was calculated using density-based spatial clustering of applications with noise [14].

#### Location Entropy

Location Entropy represented the distribution of time spent by a participant at different location clusters in one PHQ-8 interval [19] and was calculated as



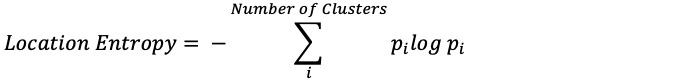



where *p*_i_ is the percentage of time spent at location cluster *i*, thus the greater the average time, the higher the Location Entropy and vice versa [19].

#### Normalized Entropy

Because the number of location clusters varies across participants and the number of clusters is positively correlated with Location Entropy [14,16,19], we also used Normalized Entropy which was given by *Normalized Entropy* = *Location Entropy* / log (*Number of Clusters*)

#### Homestay

In previous studies [13,14,16,18,19,21], each participant was assigned only one home location, which was the most visited location cluster between 12 AM to 6 AM; however, in our study, due to the long follow-up time and community-based population, participants may have more than one residential location in one PHQ-8 interval (for example, for reasons, such as traveling, business trips, or moving to a new house). Therefore, we adjusted the method of determining the residential locations. We first selected all location clusters visited at night (12 AM to 6 AM) in one PHQ-8 interval. Then, if multiple clusters were visited in the same night, the location cluster with the most location records was selected as the home location. This step partially excluded the impact of activities at night. The Homestay was the time spent at all stationary location points belonging to all home locations as the percentage of the available time in one PHQ-8 interval.

#### Residential Location Count

This new feature represented the number of residential locations. Since temporary home locations could reflect traveling [36], we used the number of residential locations in one PHQ-8 interval to reflect traveling.

### Frequency-Domain Features

**Figure 1:**
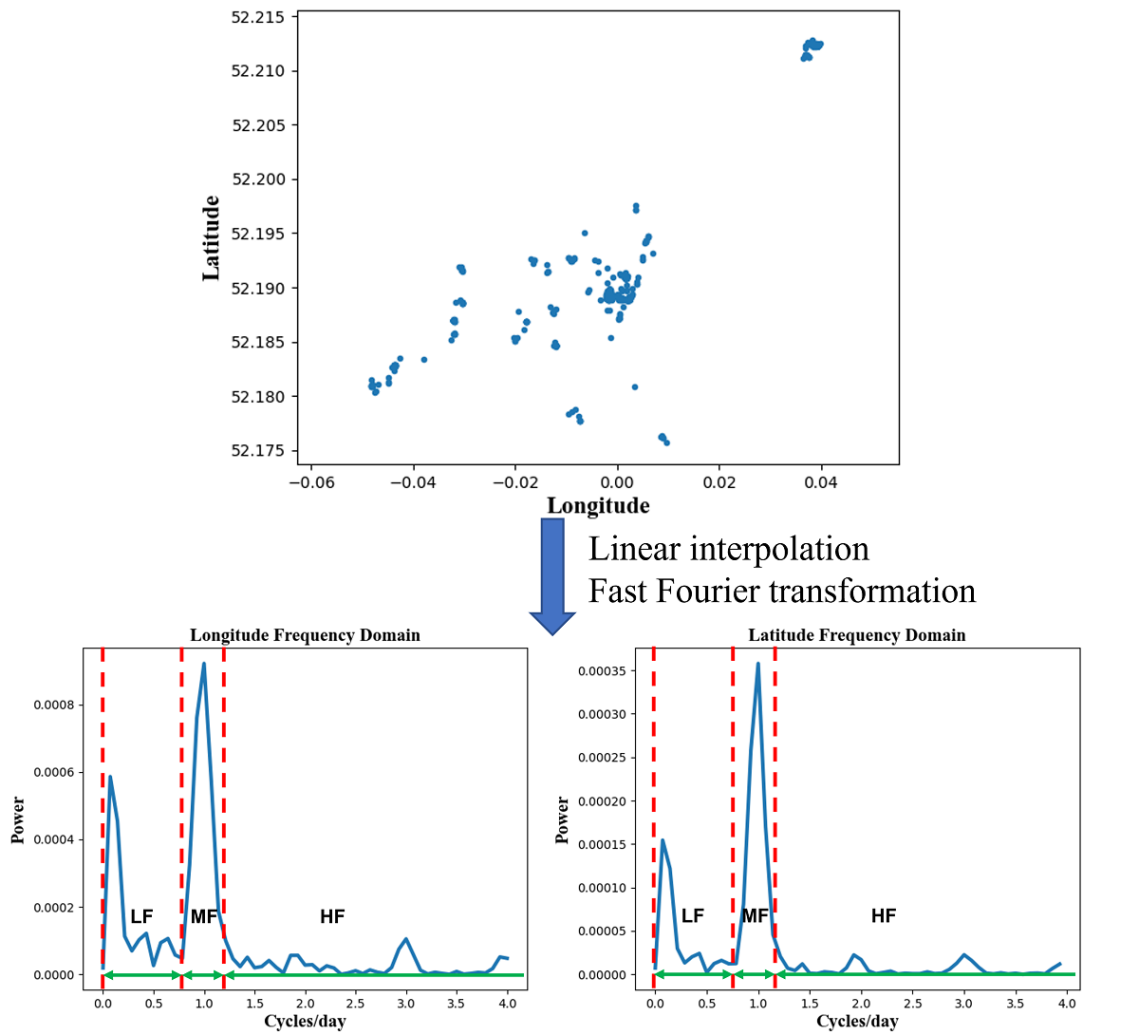
Transformation of location data from the time domain to the frequency domain. LF: low frequency (0-0.75 cycles/day); MF: middle frequency (0.75-1.25 cycles/day); HF: high frequency (>1.25 cycles/day).

People’s life rhythms (such as circadian rhythm, sleep rhythm, and social rhythm) are related to depression [37]. We proposed 3 frequency-domain features to reflect the periodicity of participants’ mobility. To compute frequency-domain features, we used linear interpolation and the fast Fourier transformation to get the spectrums of longitude and latitude data, respectively (Figure 1). The frequency axis of the spectrum was scaled in cycles per day to reflect the number of periodic patterns that occurred daily. To explore the periodic rhythms of different period lengths, we used the same frequency-domain division as in our previous publication [6], that is, frequency bands of low frequency (0 to 0.75 cycles per day), middle frequency (0.75 to 1.25 cycles per day), and high frequency (>1.25 cycles per day). The power in the middle frequency was used to represent the strength of the circadian rhythm (around 1 cycle/day) of the participant’s mobility. Likewise, the power in low frequency and high frequency represent the long-term (>1 day) periodic rhythm and short-term (<1 day) rhythm, respectively. We extracted 3 features to reflect the percentages of these 3 periodic rhythms (long-term, circadian, and short-term rhythms) in individuals’ mobility. We summed the power in the same frequency band of longitude and latitude, then divided it by the sum of the total spectral power of longitude and latitude. The formulas of these 3 features are


*Long-term Rhythm=(PSD_lon_(LF) + PSD_lat_(LF)) / (PSD_lon_(Total) + PSD_lon_(Total))*

*Circadian Rhythm=(PSD_lon_(LF) + PSD_lat_(LF)) / (PSD_lon_(Total) + PSD_lon_(Total))*

*Short-term Rhythm=(PSD_lon_(LF) + PSD_lat_(LF)) / (PSD_lon_(Total) + PSD_lon_(Total))*

where *PSD_lon_* and *PSD_lat_* represent the power spectral density of longitude and latitude, respectively, and *LF*, *MF*, *HF*, and *Total* are the low frequency, middle frequency, high frequency, and total spectral power, respectively. If the individuals’ mobility is regular, the Long-term Rhythm or Circadian Rhythm will be high, otherwise, Short-term Rhythm will be high.

### Data Analyses

**Figure 2:**
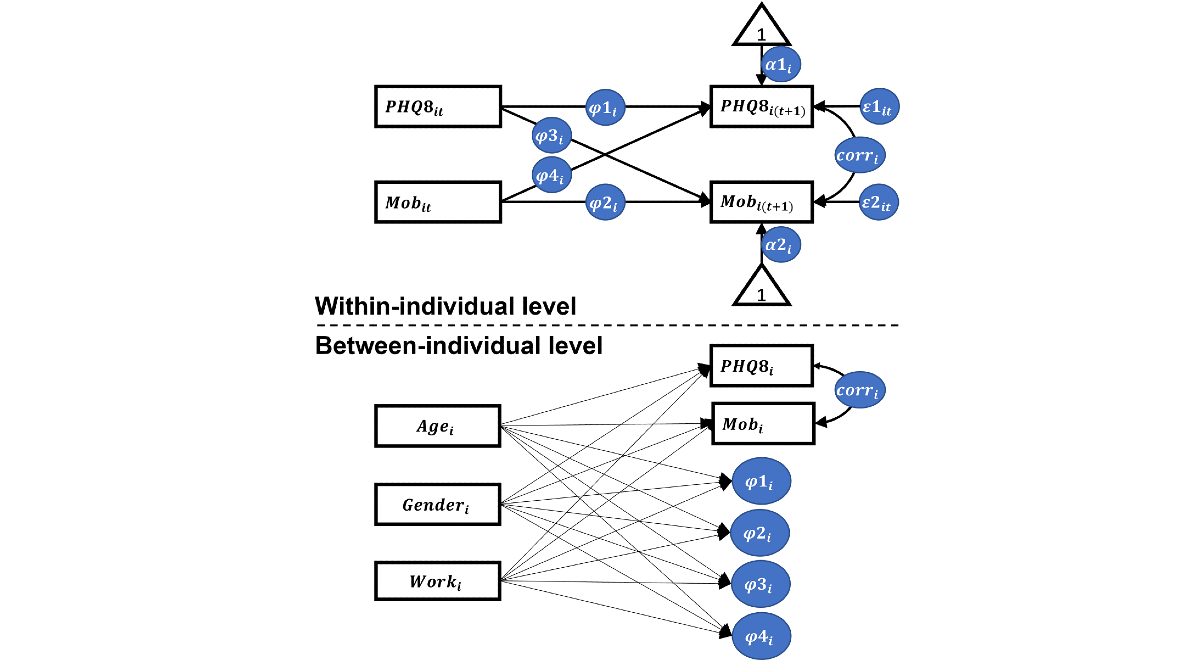
Path diagram of the vector autoregressive model. PHQ8_it_ and Mob_it_ represent the score of 8-item Patient Health Questionnaire and a mobility feature, respectively, of participant *i* at time point *t*. Age, gender, and work status were considered covariates at the between-individual level.

We used dynamic structural equation modeling to explore the relationships and the direction of relationships between mobility features and PHQ-8 scores over time. Dynamic structural equation modeling is a broad integrated framework that blends multilevel, time-series, and structural equation modeling [27,38,39] and which has shown to be particularly useful for intensive longitudinal data [38,39]. Specifically, the 2-level vector autoregressive model can estimate the lagged effects and cross-lagged effects between 2 outcome variables while considering the variability at both within-individual and between-individual levels [27,39]. The lagged effect is the impact of one variable on itself over time, which was used to represent the autoregressive nature of depressive states and mobility levels [23-25]. The cross-lagged effect is the impact of one variable on the other variable over time, which was used to explore the direction of relationships between mobility features and PHQ-8 score. In this study, we only considered the Lag-1 model (Figure 2), that is, the lagged effects and cross-lagged effects between a time point *t* and the immediately subsequent (2 weeks later) time point (*t* + 1).

We built a vector autoregressive model with each mobility feature and PHQ-8 score as outcome variables and used age, gender, and work status as covariates [40-42] at the between-individual level for adjusting individual differences. The correlations between the PHQ-8 score and the mobility feature (Figure 2) at both within-individual and between-individual levels were also estimated by the vector autoregressive model. We established a total of 11 vector autoregressive models for all mobility features. All *P* values of coefficients in vector autoregressive models and correlations were adjusted using the Benjamini-Hochberg method [43] for multiple comparisons. Findings were considered significant at adjusted *P* value <.05. Vector autoregressive models were implemented in Mplus (version 8) [44] and multiple comparison corrections were performed in R software (version 3.6.3).

## Results

### Data Summary

**Figure 3:**
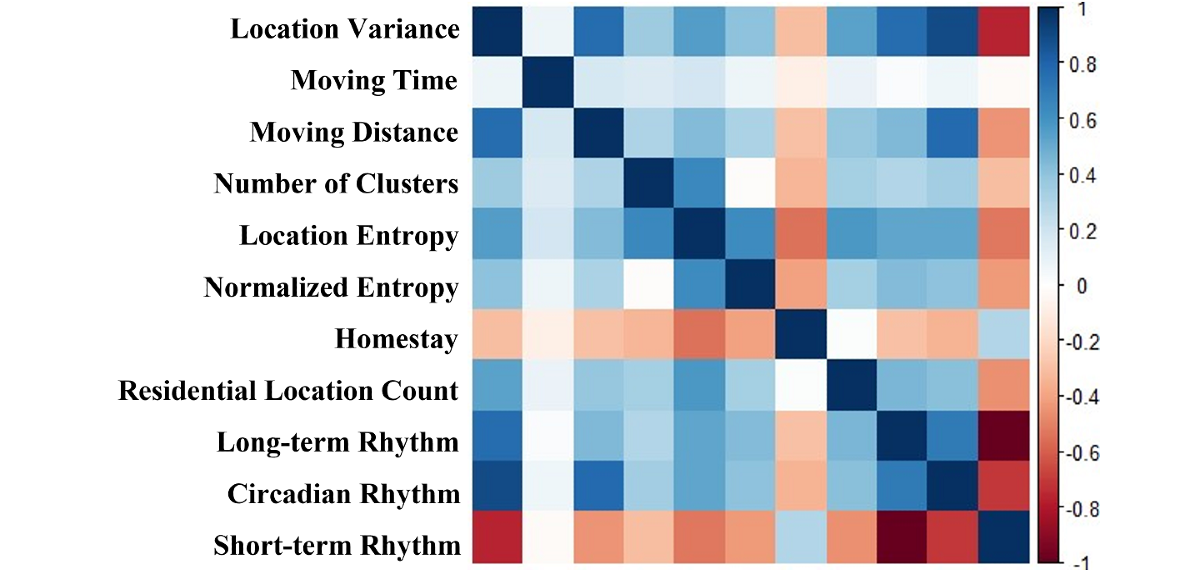
A heatmap of pairwise Spearman correlations between all 11 mobility features extracted in this paper.

The 2341 PHQ-8 intervals of 290 participants collected between November 2017 and February 2020 were included in our analysis. The sample had a median age of 50.0 (IQR 34.0, 59.0) years, with 215 (74.14%) female participants and 149 (51.38%) employed participants, with a median of 10 (IQR 5, 15) PHQ-8 scores and a median of 8.0 (IQR 3.0, 14.0) PHQ-8 intervals for each participant. The pairwise Spearman correlations between all 11 mobility features are presented in Figure 3.

### Vector Autoregressive Models

#### Correlation

Except for Moving Time (*P*=.11), all mobility features were significantly correlated with the PHQ-8 score at the within-individual level (Table 2); Homestay (ρ=0.11, *P*<.001) and Short-term Rhythm (ρ=0.07, *P*=.004) were positively correlated, while other mobility features were negatively correlated. Between individuals, Location Variance (ρ=−0.22, *P*=.04) and Moving Distance (ρ=−0.26, *P*=.04) were significantly and negatively correlated with PHQ-8 scores.

**Table 2:** Mobility features’ correlations with PHQ-8 scores at within- and between-individual levels.

Mobility feature	Within-individual level			Between-individual level	
	ρ	Adjusted *P* value	ρ		Adjusted *P* value
Location Variance	−0.10	<.001	−0.22		.04
Moving Time	0.03	.11	−0.09		.28
Moving Distance	−0.08	.002	−0.26		.04
Number of Clusters	−0.09	.001	−0.02		.44
Location Entropy	−0.15	<.001	−0.09		.22
Normalized Entropy	−0.05	.02	−0.14		.11
Homestay	0.11	<.001	0.10		.20
Residential Location Count	−0.09	.001	−0.09		.27
Long-term Rhythm	−0.07	.004	−0.17		.09
Circadian Rhythm	−0.12	<.001	−0.16		.11
Short-term Rhythm	0.07	.004	0.16		.09

#### Lagged and Cross-lagged Effects

There were significant and positive lagged effects exist in both PHQ-8 scores (φ1=0.45-0.51, *P*<.001) and mobility features (φ2=0.11-0.53, *P*<.001) (Table 3). For cross-lagged effects, PHQ-8 scores were significantly and negatively correlated with the subsequent Circadian Rhythm of mobility (φ3=−0.07, *P*<.001), while Location Entropy (φ4=−0.04, *P*=.02), Homestay (φ4=0.09, *P*=.01), and Residential Location Count (φ4=0.05, *P*=.02) were significantly correlated with subsequent PHQ-8 scores.

**Table 3:** Lagged and cross-lagged effects between mobility features and PHQ-8 scores estimated by vector autoregressive models.

Mobility feature	Lagged effects					Cross-lagged effects			
	φ1	Adjusted *P* value	φ2	Adjusted *P* value	φ3		Adjusted *P* value	φ4	Adjusted *P* value
Location Variance	0.49	<.001	0.2	<.001	−0.03		.22	0.02	.23
Moving Time	0.47	<.001	0.53	<.001	0.02		.22	0.02	.31
Moving Distance	0.48	<.001	0.38	<.001	0.03		.21	0.03	.21
Number of Clusters	0.49	<.001	0.3	<.001	0.005		.50	−0.01	.32
Location Entropy	0.47	<.001	0.22	<.001	−0.01		.33	−0.04	.02
Normalized Entropy	0.46	<.001	0.14	<.001	−0.004		.44	0.003	.45
Homestay	0.45	<.001	0.34	<.001	−0.01		.30	0.09	.01
Residential Location Count	0.51	<.001	0.11	<.001	−0.01		.34	0.05	.02
Long-term Rhythm	0.49	<.001	0.21	.001	−0.05		.06	0.001	.45
Circadian Rhythm	0.48	<.001	0.11	<.001	−0.07		<.001	0.03	.12
Short-term Rhythm	0.48	<.001	0.11	<.001	0.05		.06	−0.03	.34

#### The Influence of Individual Differences

Older and employed participants had significantly lower intercepts of the PHQ-8 score than younger and unemployed participants (Table 4). For mobility features, age was significantly and negatively correlated with Number of Clusters (γ=−0.12, *P*=.01), Location Entropy (γ=−0.18, *P*<.001), and Residential Location Count (γ=−0.16, *P*<.001), while work status was significantly correlated with most mobility features (except for Moving Time [*P*=.42] and Residential Location Count [*P*=.09]). For lagged effects, older participants had significantly lower lagged effects on Moving Distance (γ=−0.16, *P*=.02) and Homestay (γ=−0.14, *P*=.03) than younger participants. Female participants had significantly lower lagged effects on Location Entropy (γ=−0.15, *P*=.02) and Residential Location Count (γ=−0.24, *P*=.01) than male participants. Compared with unemployed participants, employed participants have significantly lower lagged effects on the PHQ-8 score (γ=−0.14, *P*=.03) and significantly higher lagged effects on Normalized Entropy (γ=0.25, *P*=.01). For cross-lagged effects, age was significantly and negatively correlated with the φ3 coefficient of Circadian Rhythm (γ=−0.49, *P*=.004) in the corresponding vector autoregressive model.

**Table 4:** Significant effects of individual difference at the between level of the vector autoregressive models. Only significant effects of at least one covariate are reported.

Characteristic			Age					Female					Employed		
			γ		Adjusted *P* value		γ			Adjusted *P* value		γ			Adjusted *P* value
**Effects on the intercept of**															
	Patient Health Questionnaire–8	−0.21		<.001		0.07			.09		−0.10			.01	
	Location Variance	−0.08		.06		0.03			.29		0.12			.01	
	Moving Distance	0.01		.47		−0.01			.40		0.07			.01	
	Number of Clusters	−0.12		.01		0.02			.36		0.09			.03	
	Location Entropy	−0.18		<.001		0.01			.40		0.20			<.001	
	Normalized Entropy	−0.09		.09		−0.01			.45		0.26			<.001	
	Homestay	0.01		.32		0.03			.16		−0.15			<.001	
	Residential Location Count	−0.16		<.001		0.04			.17		0.06			.09	
	Long-term Rhythm	−0.07		.07		0.02			.34		0.14			.01	
	Circadian Rhythm	−0.07		.08		0.06			.10		0.13			<.001	
	Short-term Rhythm	0.10		.06		−0.06			.13		−0.16			<.001	
**Effects on the lagged effect of**															
	Patient Health Questionnaire–8	0.01		.47		−0.07			.13		−0.14			.03	
	Moving Distance	−0.16		.02		−0.04			.31		−0.08			.06	
	Location Entropy	−0.01		.46		−0.15			.02		0.02			.38	
	Normalized Entropy	0.09		.19		−0.19			.05		0.25			.01	
	Homestay	−0.14		.03		−0.09			.13		0.05			.27	
	Residential Location Count	0.01		.48		−0.24			.01		−0.04			.36	
**Effects on the cross-lagged effect of**															
	Circadian Rhythm (φ3)^a^	−0.49		.004		0.01			.48		0.164			.25	

^a^φ3 represents the effect of the Patient Health Questionnaire–8 on the subsequent mobility feature.

## Discussion

### Principal Findings

This study provides a comprehensive understanding of the relationships and the direction of the relationships between depressive symptom severity and phone-measured mobility over time by using dynamic structural equation modeling on a large longitudinal data set and considering correlations at both individual and population levels, lagged effects (the autoregressive nature over time), cross-lagged effects (direction of the relationships over time), and the influences of individual differences (demographic characteristics).

Most mobility features extracted in this paper were significantly correlated with the PHQ-8 score at the within-individual level (Table 2), which indicated that, for a participant, the higher the severity of depressive symptoms, the lower mobility. This is consistent with both past survey-based [9] and phone-based studies [18,19]. These findings reaffirmed that the link between depressive symptom severity and mobility can be captured by mobile phones. However, many of the mobility features’ correlations with PHQ-8 score were not significant at the between-individual level, possibly due to the significant effects of individual differences (age and work status) on both PHQ-8 score and mobility features (Table 4). Notably, features of Location Variance (ρ=−0.22, *P*=.04) and Moving Distance (ρ=−0.26, *P*=.04) were still significantly correlated with PHQ-8 score at the between-individual level, which indicated these features are relatively robust for reflecting depressive symptom severity in the whole population. Compared with the results of previous phone-based studies, our results showed that population diversity affects correlations between mobility features and the depression score. Most mobility features were significantly correlated with depression scores in student-based studies [16,18], while several features lost their significance in a community-based population with a wide age distribution [19]. These findings indicated that individual differences need to be considered during exploring relationships between depression and mobility.

PHQ-8 score and mobility features both had significant and positive lagged effects (Table 3), indicating that the autoregressive nature of individuals’ depressive states [24] and movement habits [25] could be captured by mobile phones. For the direction of relationships over time, we found 3 mobility features significantly correlated with the subsequent PHQ-8 score. Specifically, increases in PHQ-8 score are probably preceded by one or more following changes in the mobility: (1) lower average time spent at different places (Location Entropy), (2) more time at home (Homestay), and (3) more traveling (Residential Location Count). Conversely, change in PHQ-8 score was significantly and negatively correlated (φ3=−0.07, *P*<.001) with the subsequent circadian rhythm measured by location data. The findings of a recent study [22] showed changes in several mobility features were associated with subsequent depression changes, but not vice versa. The differences in populations and applied methods could be potential reasons for the slightly inconsistent results. Both our study and that study [22] have shown that the changes in mobility prior to changes in depressive symptom severity can be captured by mobile phones. An interesting finding is that the number of residential locations was positively correlated (φ4=0.05, *P*=.02) with the subsequent PHQ-8 score (Table 3), which is opposite to their negative correlation (ρ=−0.09, *P*=.001) at the within-individual level (Table 2). As the number of temporary residential locations could reflect traveling [36], this finding indicated that traveling may reduce the current depressive symptoms but may worsen some existing depressive feelings. This finding may provide insight into a phenomenon called “post-travel depressed feelings [45,46].” The causes of “post-travel depressed feelings” are fatigue from trips, the shock of re-entry of ordinary life, and jet lag [46,47].

For influences of individual differences on the levels of depressive symptom severity and mobility, we found that PHQ-8 scores tended to be lower in participants who are older or have jobs, which can be expected because previous survey-based studies have shown that depression is negatively correlated with age, and the unemployment rate in the depressed population is high [40-42]. Gender was not significantly correlated with the PHQ-8 score (γ=0.07, *P*=.09) in our population, possibly due to all participants in our study having at least one diagnosis of depression in recent 2 years [26], which may reduce the link between gender and depressive symptom severity. For the effects of demographic characteristics on mobility features, we found that the mobility in older participants or participants without jobs tended to be lower, which is also expected. For influences of individual differences on the lagged and cross-lagged effects, we found the participants with jobs had lower autocorrelation of the PHQ-8 score, indicating more depressive symptoms severity changes over time in employed participants than unemployed participants. Female participants, older participants, and unemployed participants tended to have lower autocorrelations of some mobility features than male participants, young participants, and employed participants, which indicated that variabilities of mobility over time were larger in these participants. For influences of age on cross-lagged effects, the impact of changes in PHQ-8 score on the subsequent circadian rhythm for older participants was significantly lower than that of young participants (γ=−0.49, *P*=.004), indicating that the mobility rhythm of the older participants is affected by depressive symptoms for a shorter period than the young participants.

We proposed 3 frequency-domain features to reflect the periodic characteristics of individuals’ mobility (Figure 1). They were all significantly correlated with the PHQ-8 score at the within-individual level. Higher values of Long-term Rhythm and Circadian Rhythm represent more regular movement and activity, which were correlated with lower depressive symptom severity. Notably, Circadian Rhythm had the strongest correlation (ρ=−0.12, *P*<.001) among these 3 features, and it had significant cross-lagged effect (φ3=−0.07, *P*<.001) with the preceding PHQ-8 score. These findings demonstrated that the frequency-domain of location data can provide some additional information for evaluating depressive symptom severity in future research.

### Limitations

We obfuscated the raw location data due to privacy issues. Therefore, we did not have access to contextual information, which may mean some information was lost. Another limitation is that we only used the Lag-1 vector autoregressive models. We did not use high-order vector autoregressive models because we wanted to make our preliminary model simple to allow easier explanation and to avoid convergence problems in the procedure of coefficient estimations. We will attempt high-order vector autoregressive models in future research when we have more data without the impact of the COVID-19.

We chose to build 11 dynamic structural equation modeling models, one for each mobility feature. Since each mobility feature has a specific meaning, the bivariate model can better explain changes of the feature before and after the changes in PHQ-8 scores indicating the longitudinal relationships. We attempted multivariate dynamic structural equation modeling with all mobility features, but the model failed to converge, possibly due to the multicollinearity between mobility features and complexity of the model. As all mobility features were devised for describing characteristics of individuals’ mobility, there were high correlations between mobility features (Figure 3). In future research, we plan to solve the multicollinearity in the multivariate model through further feature engineering and feature selection methods or by using other multivariate time series models which are robust to multicollinearity [48].

### Conclusions

This study provides initial evidence of the relationship and the direction of the relationship between depressive symptom severity and phone-measured mobility over time. We found several mobility features affected depressive symptom severity, while changes in the depression score were associated with the subsequent periodic rhythm of mobility. These mobility features have the potential to be used as indicators for assessing depression risk in future clinical applications, which could provide timely suggestions for both people with depression risk (eg, encouraging to attend more activities) and physicians (eg, early interventions). This work may provide support for remote mental health monitoring practice in real-world settings.

## Abbreviations

NIHR: National Institute for Health Research
NHS: National Health Service
PHQ-8: 8-item Patient Health Questionnaire
RADAR-CNS: Remote Assessment of Disease and Relapse–Central Nervous System
